# Pericapsular Nerve Group Block Plus Lateral Femoral Cutaneous Nerve Block vs. Fascia Iliaca Compartment Block in Hip Replacement Surgery

**DOI:** 10.3390/jcm14030984

**Published:** 2025-02-04

**Authors:** Francesco Vetrone, Francesco Saglietti, Andrea Galimberti, Angelo Pezzi, Michele Umbrello, Giuseppe Cuttone, Luigi La Via, Luigi Vetrugno, Cristian Deana, Alessandro Girombelli

**Affiliations:** 1 Department of Anesthesiology, Pain Medicine and Intensive Care Unit, Policlinico di Monza, 20900 Monza, Italy; francesco.vetrone@asst-nordmilano.it; 2 Department of Emergency, Anesthesia and Critical Care, Division of Anesthesiology, Santa Croce and Carle Hospital, 12100 Cuneo, Italy; saglietti.md@gmail.com; 3 Department of Anesthesiology and Critical Care Medicine, ASST Nord Milano, Bassini Hospital, via Gorki, 50, 20092 Cinisello Balsamo, Italy; andrea.galimberti@asst-nordmilano.it (A.G.); angelo.pezzi@asst-nordmilano.it (A.P.); 4 Department of Intensive Care and Anesthesia, ASST Ovest Milanese, Ospedale Civile di Legnano, 20025 Legnano, Italy; michele.umbrello@asst-ovestmi.it; 5 Faculty of Medicine and Surgery, Kore University, 94100 Enna, Italy; giuseppe.cuttone@hotmail.it; 6 Department of Anesthesia and Intensive Care 1, University Hospital Policlinico “G. Rodolico-San Marco”, 95123 Catania, Italy; luigilavia7@gmail.com; 7 Department of Anesthesiology, Critical Care Medicine and Emergency, SS. Annunziata Hospital, 66100 Chieti, Italy; luigi.vetrugno@unich.it; 8 Department of Medical, Oral and Biotechnological Sciences, University of Chieti-Pescara, 66100 Chieti, Italy; 9 Department of Anesthesia and Intensive Care, Health Integrated Agency of Friuli Centrale, 33100 Udine, Italy; 10 Department of Anesthesiology, Intensive care and Emergency Medicine, Division of Anesthesiology, EOC, Ospedale Regionale di Lugano, 69000 Lugano, Switzerland; alessandro.girombelli@asst-nordmilano.it

**Keywords:** hip replacement, locoregional anesthesia, pain, postoperative recovery, opioids, ultrasound

## Abstract

**Background**: Optimal pain control with limited muscle weakness after total hip arthroplasty (THA) is paramount for a swift initiation of physical therapy and ambulation, thus expediting hospital discharge. FICB (Fascia Iliaca Compartment Block) has been recommended because it offers pain control with a low risk of motor block. PENG (Pericapsular Nerve Group) block with LFCN (Lateral Femoral Cutaneous Nerve) has been proposed as an alternative that offers comparable pain control with a lower risk of motor block; however, evidence is limited. We aimed to investigate the degree of muscle weakness and pain control with PENG + LFCN. **Methods**: Patients undergoing elective THA from November 2022 to October 2023 have been retrospectively analyzed. The degree of quadriceps femoris muscle paresis was assessed with the MRC scale at 6 h postoperatively. Secondary outcomes: NRS score at 6, 12, and 24 h, total opioid consumption, and time to first rescue opioid. **Results**: In total, 80 patients were included in the study, 57 received PENG + LFCN, and the remaining 23 received FICB. PENG + LCFN resulted in a higher MRC at 6 h (4 [4; 5] vs. 3 [2; 4] *p* = 0.0001) and better pain control (mean difference [95% CI] at 6 h, 0.93 [0.14; 1.72], at 12 h, 0.47 [−0.49; 1.43], and at 24 h, 0.39 [0.25; 1.2], *p* = 0.0006). Less PRN opioids were requested in the PENG + LFCN vs. FICB groups (7.5 [0; 15] MME vs. 60 [40; 80], *p* = 0.001). **Conclusions**: PENG + LFCN was associated with less muscle weakness, better pain control, and less rescue opioids in patients undergoing elective THA. A larger prospective study is needed to confirm this finding.

## 1. Introduction

Total hip arthroplasty (THA) is one of the most frequently performed orthopedic procedures worldwide [1,2,3]. It has been shown to have the potential to significantly improve quality of life by alleviating joint pain and restoring proper mobilization [4]. However, due to the complex innervation of the hip [5], effective perioperative pain management is challenging, making it difficult to achieve optimal analgesia while minimizing muscle weakness [6]. It is well-documented that the incidence of both acute and chronic postoperative pain following THA is high, which can often hinder early physical rehabilitation [7,8,9]. Early postoperative interventions, such as physical therapy, ambulation, and optimal pain control, after THA are pivotal for minimizing the risk of complications, such as deep vein thrombosis and postoperative delirium [10,11,12,13]. As such, these perioperative interventions play a crucial role in achieving a shorter hospital stay and contribute to decreasing as much as possible the mortality rates associated with this surgical procedure, thereby improving overall patient outcomes [14,15,16,17,18,19].

An optimal anesthetic strategy for hip surgery should balance adequate pain control with the least postoperative muscle weakness, which is essential for prompt initiation of physical therapy, as mentioned earlier.

Pain management strategies for THA include a variety of approaches, each with its own set of advantages and challenges. One of the first attempts to provide good analgesia beyond the intraoperative period is represented by epidural analgesia. This technique was initially performed using a continuous infusion of long-lasting local anesthetic, such as bupivacaine or ropivacaine, delivered via an elastomeric pump or an infusion pump. The continuous infusion resulted in effective pain control, but the incidence of lower limb motor weakness is debated in the available studies [20,21]. An improvement in this regional anesthesia technique was implemented with the use of patient-controlled epidural analgesia. By connecting the epidural catheter to an infusion pump equipped with a “demand” button, the patient could self-administer a bolus dose of epidural analgesia whenever the pain level rose [22]. There is quite a degree of heterogeneity in the choice of the volume of the bolus and the speed of maintenance infusion [23]. A combination of a bolus dose plus a continuous infusion to ensure a basal level of analgesia is often found in the literature. The continuous infusion might theoretically represent a problem by sustaining a degree of motor block that might persist until the infusion speed is reduced and the effect of the local anesthetic wears off, but, to date, no study supports this hypothesis [21,22]. A novel approach could be the use a programmed intermittent epidural bolus (PIEB) technique where a bolus dose of local anesthetic is administered at fixed time intervals combined with a rescue bolus and the possibility to prescribe a maintenance infusion; a recent study by Kang et al. suggests how this approach might provide superior analgesia compared to continuous epidural infusion [24]. Another possibility is the use of adjunctive agents to enhance the effectiveness of local anesthetics. Opioids are the most common choice of adjuncts as they improve pain control without increasing the dose and concentration of the local anesthetic, thus limiting the risk of motor block [25]. Epidural analgesia is not without risk, as it is associated with a non-negligible risk profile that encompasses epidural hematoma, dural puncture, nerve damage, prolonged motor block, and delayed hospital discharge. Intrathecal opioids, administered alongside local anesthetics during spinal anesthesia, have been described in the literature. The most common opioids used are morphine, fentanyl, and sufentanil, with a wide range of dosages reported. This approach shares most of the periprocedural complications seen with epidural analgesia. The pain seems to be adequately controlled in the first 33 h postoperatively, with a risk of pain recurrence beyond this time point [26].

Alternatively, a number of extra-axial analgesic techniques include several peripheral nerve blocks, such as Femoral Nerve Block (FNB), Lumbar Plexus Block (LPB), Lateral Femoral Cutaneous Nerve Block (LFCN Block), Quadratus Lumborum Block (QLB), and Fascia Iliaca Compartment Block (FICB). Lastly, local infiltration by the surgeon (Local Infiltration Analgesia (LIA)) has been described in the literature and is often employed as a comparator for regional anesthesia techniques [27,28].

The Femoral Nerve Block (FNB) has been shown to reduce postoperative pain by decreasing the need for as-needed opioid therapy [29]. However, this particular nerve block is associated with delayed ambulation and physical therapy as it inevitably results in a motor blockade of the quadriceps femoris muscle [30].

The Lumbar Plexus Block (LPB) is another effective option for pain control, but it is a more invasive technique with a higher risk of complications. This is due to its deep anatomical location, its close proximity to vital structures, such as the kidneys and the aorta, and the fact that it is often performed without ultrasound guidance. Additionally, LPB can lead to prolonged motor block, delayed recovery, and an increased risk of falls, while also posing potential risks of vascular injury, nerve injury, and neurotoxicity give the high vascularization of the psoas muscle [31,32].

The Quadratus Lumborum Block (QLB) is a fascial plane block that has shown promise in reducing acute postoperative pain. Given the novelty of this approach, more research is needed to confirm its effectiveness in this particular population [33,34].

The Lateral Femoral Cutaneous Nerve Block (LFCN Block) has recently been investigated. This block provides analgesia to the lateral aspect of the thigh but offers no coverage for articular pain. As such, this block must be administered in conjunction with a second block that addresses articular pain, such as the PENG Block or the Fascia Iliaca Compartment Block. This combination of peripheral nerve blocks has been shown to significantly reduce movement-related pain [33,35,36].

While there is no definitive consensus in the literature regarding the optimal analgesic technique, a substantial body of evidence supports the use of the FICB [37]. The PROSPECT and ICAROS guidelines endorse the FIC Block as the preferred anesthetic technique due to its ease of execution and reliable analgesia [33,38].

However, it must be noted that FICB has several shortcomings. As the evidence suggests, FICB fails to provide satisfactory pain relief to the medial hip joint and the crural region [39], and it is associated with lingering quadriceps motor block in as many as 70% of cases. This muscle weakness lasted well over 6 h after the block was performed. However, this side effect may significantly delay the initiation of early rehabilitation, but it did not result in an increase in the number of falls [40,41].

The Pericapsular Nerve Group Block (PENG) is a relatively novel peripheral nerve block technique [42] that has emerged as a promising technique for addressing hip-related pain ranging from hip fractures to elective hip replacement surgery to chronic osteoarthritis pain. This block has been shown to relieve articular pain and provide adequate postoperative analgesia for THA while preserving quadriceps muscle strength, assessed in terms of both range of quadriceps muscle activity and time to first walk [43,44,45,46,47,48].

The effectiveness of the PENG Block lies in its ability to block the articular rami of the femoral nerve, the obturator nerve, and the accessory obturator nerve that are responsible for innervating the anterior hip capsule [43,49]. Because the density of mechanoreceptors and nociceptors in the posterior capsule is significantly lower, the posterior portion of the joint capsule, innervated by the superior gluteal, the inferior gluteal, and the sciatic nerves, holds less clinical significance for pain management [50]. As a result, specific analgesic interventions in this area are unnecessary. For this reason, it could be reasonable to say that the PENG Block has the ability to block all of the nerves involved in the generation of post-surgical articular pain.

The main limitation of this technique is its inability to treat the pain arising from the surgical incision site for THA performed via the direct anterior approach (DAA) because the innervation of the antero-lateral aspect of the thigh is provided by the Lateral Femoral Cutaneous Nerve (LFCN). Thus, an additional treatment, such as local infiltration of anesthetic (LIA) by the surgeon at the end of the procedure, is required to completely cover postoperative pain. Several case reports and case series have suggested a different approach to this problem. By combining PENG with the LFCN Block, an interesting and effective alternative to FICB and LIA can be offered to hip replacement surgery patients. This analgesic choice offers comparable pain control with a substantially lower risk of motor block [51,52,53,54,55,56,57,58,59].

A prior pilot study conducted by our group explored this hypothesis by retrospectively comparing the combination of PENG + LFCN Block with FICB, with the primary outcome being the lowest postoperative numerical pain rating score (NRS) [60]. Secondary outcomes included the incidence of residual quadriceps motor block and the requirement for rescue opioids, measured in morphine milligram equivalents (MME). The results indicated that the PENG + LFCN Block was associated with significantly lower NRS scores and reduced postoperative MME consumption compared to FICB. However, there was no significant difference in the degree of residual motor block between the two groups. Given that residual motor block, particularly in the quadriceps, is a secondary outcome with substantial clinical implications for early rehabilitation, we decided to conduct a follow-up study to further investigate this issue. The goal of this new study is to retrospectively compare the PENG + LFCN Block with FICB, specifically focusing on determining which technique results in the least degree of quadriceps muscle weakness, as this has direct implications for early mobilization and recovery after THA.

## 2. Materials and Methods

The current trial was registered on ClinicalTrials.gov (Study ID NCT06342102) on 2 April 2024. After obtaining ethics committee approval (“Comitato Etico Territoriale Lombardia 3”) in June 2024, Number 4286_24/06/2024, an historical cohort study was conducted among all patients who had THA from November 2022 to October 2023 at a tertiary hospital center in the metropolitan city of Milan.

The inclusion criteria were elective THA for non-traumatic hip disease, age over 18 years, complete clinical chart, including type of peripheral nerve block performed, and signed consent form for spinal anesthesia and peripheral nerve block.

The exclusion criteria were preoperative opioid therapy, having received a peripheral nerve block other than PENG + LFCN or FICB, having received general anesthesia, incomplete chart, documented muscle weakness and deviation from the established post-operative analgesia protocol, and any traumatic hip surgery.

The patients that underwent hip replacement surgery were identified by filtering the ICD codes marked in the hospital’s operating room digital charting program (Hopera–Hospital Operating Procedures Editing, Retrieval and Administration–designed by Sysline S.p.A, Milan, Italy,. and Ormaweb by Dedalus Healthcare Systems Group s.p.a, Florence, Italy). The following ICD codes were chosen for the search:

ICD 715.15 “primary hip arthrosis” (79 patients were found);

ICD 715.25 “secondary hip arthritis” (3 patients were found);

ICD 715.35 “unspecified primary or secondary hip arthrosis” (3 patients were found);

ICD 715.95 “unspecified generalized or localized hip arthrosis” (1 patient was found);

ICD 733.42 “aseptic necrosis of the head and neck” (7 patients were found).

The peripheral nerve blocks were performed immediately after spinal anesthesia to minimize patient discomfort.

The PENG Block was performed using an in-plane technique under real-time ultrasound guidance with a 2–5 MHz curvilinear probe of the Ecube i7 system (Alpinion, Biolive Group, Seoul, Republic of Korea), with an 80 mm echogenic needle (SonoPlex II PAJUNK^®^, Geisingen, Germany) inserted from lateral to medial.

The procedure began with skin disinfection using 2% Chlorhexidine, ensuring sterile conditions. The needle tip was carefully advanced to the osseous plane between the psoas tendon and the pubic ramus. After negative aspiration and after confirming correct needle placement with a 1 mL NaCl 0.9% test injection, 20 mL of 0.5% ropivacaine was injected to effectively block the articular rami of the femoral nerve, the obturator nerve, and the accessory obturator nerve. The correct placement of the block was routinely verified by monitoring the spread of fluid within the plane and the displacement of the psoas tendon.

The LFCN Block was performed following the PENG Block. A linear ultrasound probe with a frequency range of 7.5–12 MHz was used to guide the procedure with an in-plane approach, allowing for precise localization of the Lateral Femoral Cutaneous Nerve (LFCN). After skin disinfection, an 80 mm echogenic needle (SonoPlex II PAJUNK^®^, Geisingen, Germany)) was inserted from lateral to medial. The choice of this needle length was justified because this block was performed after the PENG Block, and changing the needle to a 50 mm long needle was deemed unnecessary and wasteful. After negative aspiration and confirming correct needle placement with a 1 mL NaCl 0.9% test injection, a total of 10 mL of 0.5% ropivacaine was injected near the nerve, targeting the fat-filled flat tunnel (FFFT) located in the plane between the tensor fasciae latae muscle (TFLM) and the sartorius muscle (SaM), typically 1–2 cm medial and inferior to the superior anterior iliac spine (ASIS). Proper execution of the block was confirmed by visualizing the spread of the local anesthetic in the described plane (i.e., FFFT) around the LFCN, superficial to the SaM.

For the Fascia Iliaca Compartment Block (FICB), the procedure was performed with in-plane ultrasound guidance using a 50 mm ultrasound-compatible needle. A linear ultrasound probe with a frequency range of 7.5–12 MHz was used to visualize the relevant anatomical structures, including the fascia iliaca, superficial to the iliopsoas muscle (IpM), medially to the sartorius muscle (SaM), and in proximity to the femoral artery (FA), the femoral vein (FV), and the femoral nerve (FN).

The goal was to position the needle tip in the plane between the fascia iliaca and the iliopsoas muscle, approximately at the lateral third of the line connecting the anterior superior iliac spine (ASIS) to the pubic tubercle. After skin disinfection, the needle was carefully advanced toward the target site. After negative aspiration and confirming correct needle placement with a 1 mL NaCl 0.9% test injection, a total of 20 mL of 0.5% ropivacaine was injected into the fascia iliaca compartment. It is critical to monitor the spread of the local anesthetic (LA) using ultrasound visualization to ensure proper execution of the block and to assess its success. The LA should spread in a medial-to-lateral direction from the injection site, extending toward the femoral nerve medially and laterally toward the iliac spine, underneath the sartorius muscle.

Spinal anesthesia was performed after skin disinfection, with the patient lying in the lateral decubitus position. To do so, 0.5% hyperbaric bupivacaine at 0.05 mg/height in cm plus intrathecal sufentanil 5 mcg were administered through a 27 G Sprotte spinal needle.

All patients received the same multimodal analgesia regimen, which included acetaminophen (3 g daily) and ibuprofen 400 mg every 8 h, unless contraindicated. This regimen was routinely initiated during the intraoperative phase. For patients with suboptimal postoperative pain control, rescue oral opioid medications, either oxycodone/naloxone or tapentadol, were provided at the minimum necessary dose, as recommended by international guidelines (CDC) [61]. These opioids were administered when the Numerical Rating Scale (NRS) exceeded 5. According to protocol, the nursing staff and physical therapist attempted to mobilize all patients on the first postoperative day.

All of the surgeries were conducted in strict accordance with the ERAS (Early Recovery After Surgery) protocols by a dedicated and experienced team of surgeons and anesthesiologists. To ensure consistency and eliminate variability in the procedural approach, the same surgical technique (THA with the direct anterior approach, DAA) was employed for all patients. Additionally, a uniform analgesic protocol was followed for all participants, ensuring standardization in pain management throughout the study.

Comprehensive data were systematically collected from clinical records and digitally organized using an Excel spreadsheet (Microsoft Office, Redmond, WA, USA). Demographic and clinical characteristics, including age, sex, weight, height, body mass index (BMI), and the American Society of Anesthesiologists (ASA) clinical classification, were documented to provide a detailed profile of the study population. To assess the impact of the different analgesic strategies, we collected data on the type of peripheral block used for each patient and the incidence of quadriceps femoris muscle weakness, assessed using the Medical Research Council Manual Muscle Test Scale (MRC) at 6 h postoperatively (scores ranged from 0, indicating no contraction, to 5, indicating normal muscle strength).

For secondary outcomes, the Numerical Rating Scale (NRS) at rest was recorded at 6, 12, and 24 h after surgery. Data on the time to the first PRN (pro re nata, i.e., as-needed) opioid request and the total PRN opioid dose administered were also collected. This allowed for the assessment of the effectiveness of the analgesic interventions and the evaluation of opioid consumption. Any complications, such as nausea, vomiting, hypo- or paresthesia, hypotension, or falls, were meticulously documented to ensure close monitoring of patient safety and any potential side effects related to anesthesia or analgesia.

### Statistical Analysis

Categorical variables were presented as absolute frequencies (*n*) and percentages (%). Given the limited number of observations, continuous variables were expressed as the median and the interquartile range (IQR), defined by the 25th and 75th percentiles. This approach was chosen to accurately summarize the distribution of the data. Data analysis was performed using STATA 14.0 (StataCorp LLC, College Station, TX, USA).

The following statistical tests were applied to assess differences between groups:

- Fisher’s exact test for categorical variables, used to determine if there were non-random associations between the groups.

- Student’s *t*-test or its non-parametric counterpart, the two-sample Wilcoxon rank-sum test, depending on the data distribution. These tests were used to compare continuous variables, with the choice of test being contingent on whether the data followed a normal distribution or not.

- Two-way ANOVA for repeated measures was used to assess the impact of both the type of peripheral block and the time on the outcomes by comparing the variables between the two cohorts at multiple time points. The type of block (an inter-subject variable) and the time (an intra-subject variable) were treated as independent categorical factors. The interaction between these two variables (i.e., the combined effect of block type and time) was also incorporated into the model. Statistical significance for the intra-subject factor was adjusted using the Greenhouse–Geisser method to account for any potential violations of sphericity. If a statistically significant interaction was identified between variables, the Siegel–Tukey Test for multiple interactions was employed to further investigate the nature of the relationship between the factors.

Three distinct *p*-values were reported to provide a comprehensive assessment of the data:

- *p* (block): This represents the group effect, which is used to compare NRS scores between the different cohorts or sub-populations.

- *p* (time): This represents the time effect, which is used to compare NRS scores across different time points.

- *p* (block × time): This combined effect takes into account the interaction between the group and time, providing insight into how the relationship between block type and time affects the outcomes.

A *p*-value of <0.05 was considered statistically significant for all tests.

## 3. Results

**Figure 1:**
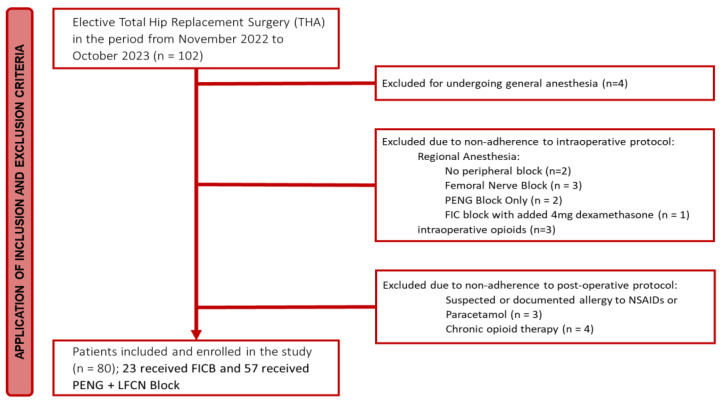
Study flow-chart.

A total of 102 patients underwent elective total hip replacement surgery during the study period. After applying the exclusion criteria, a total of 22 patients were excluded. Specifically, two patients were excluded because they received general anesthesia, two had no peripheral block clearly reported on the anesthesiology chart, five received a different peripheral block from those specified in the study (two received a Femoral Nerve Block and three received the PENG Block only), one patient received a peripheral block with added dexamethasone, three patients were excluded due to non-adherence to the protocol for intraoperative opioid administration, four were on chronic opioid therapy, and three had suspected/documented allergy to NSAIDs or paracetamol (Figure 1).

The study population had no difference in baseline characteristics (Table 1).

**Figure 2:**
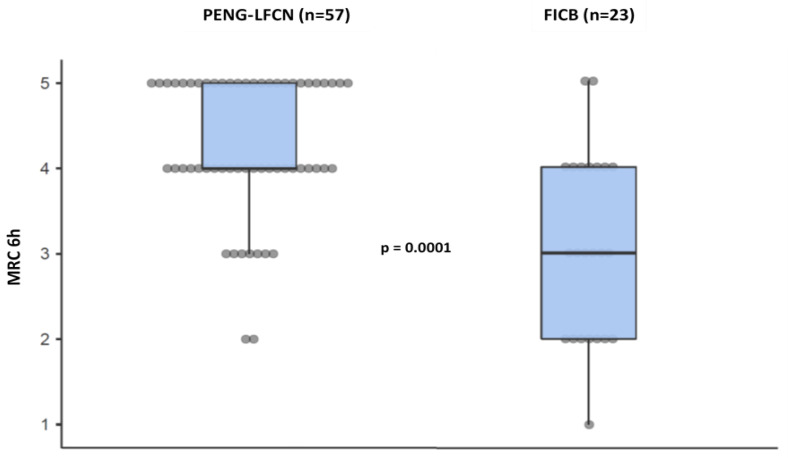
Evaluation of recovery of quadriceps femoris muscle strength six hours after block. Legend. PENG: Pericapsular Nerve Group. LFCN: Lateral Femoral Cutaneous Nerve. FICB: Fascia Iliaca Compartment Block. MRC: Medical Research Council Scale for Muscle Strength.

The PENG + LFCN group had a significantly higher MRC score at 6 h compared to the FIB group (4 [4; 5] vs. 3 [2; 4] *p*-value = 0.0001), as shown in Figure 2.

Amongst the secondary outcomes, patients receiving PENG + LCFN had a statistically significant lower reported NRS score at all time points, as shown in Table 2 and Table 3.

Rescue opioid requirements were considerably lower in the PENG + LFCN group than the FICB group (7.5 MME vs. 60 MME, respectively, *p*-value = 0.0001). Moreover, in the former group, the first rescue opioid was administered at a later time point compared to FICB (11.5 h [8; 12] vs. 4 h [4; 8], *p*-value = 0.0001). Lastly, only 54.5% of the patients in the PENG + LFCN group required rescue opioid medication, compared to 91.3% in the FIB group (*p*-value 0.002).

No significant adverse events, such as nausea, vomiting, or falls, were reported for either group.

## 4. Discussion

The PENG + LFCN group was associated with less muscle weakness compared to the FICB group. Specifically, the primary outcome of limb motility recovery reached statistical significance in favor of PENG + LFCN, with a one-point higher MRC compared to FIB (four vs. three). This result is also of clinical significance, as the ability to move the limb against gravity and resistance (i.e., MRC = 4) could allow for physical rehabilitation as early as the first 6 h after surgery, therefore reducing the risks of thromboembolic events and chronic postoperative pain.

Amongst the secondary outcomes, the reported value of pain was lower in the PENG + LFCN group in all three time intervals compared to FICB. Although the extent of reduction in the NRS score (1 point) might seem modest and of little clinical significance, several published articles acknowledge an NRS ≤ 1 as the threshold for clinical relevance of the anesthetic technique under consideration [62,63].

Given the importance of effective pain control in the context of postoperative management to allow for early rehabilitation, this secondary outcome appears to be important.

The secondary outcome of the total dose of rescue opioids administered yielded an interesting result, as the FIC group received, on average, more than 40 MME compared to 7.5 MME for the PENG group.

This outcome would seem to imply that PENG + LFCN Block could limit perioperative opioid consumption and thus reduce the number of side effects associated with long-term opioid use (i.e., reduce the risk of opioid addiction), as suggested by many international guidelines [61].

This finding has been described before by our group in a previous pilot study [60]. Given the aforementioned benefits in terms of opioid sparing, it could be interesting to investigate this finding with a prospective study focused on perioperative opioid consumption.

It should, nonetheless, be emphasized that both approaches demonstrated optimal pain control, bringing further evidence in support of a multimodal analgesic protocol that includes regional anesthesia techniques instead of a purely systemic approach to analgesia, as currently suggested by international guidelines.

A recent randomized controlled trial has been conducted on this topic; amongst the 92 subjects scheduled for elective one-sided hip replacement with the posterolateral approach under general anesthesia, the 46 who received PENG + LFCN Block had a significantly shorter time to first walk, a higher degree of hip flexion at all postoperative time points, and the least limb weakness in the first 6 h postoperatively compared to those who received suprainguinal FICB [64,65].

While the results of this study are mostly in line with our findings, it must be noted how a direct comparison is limited by the choice of general anesthesia instead of spinal anesthesia, the posterolateral surgical approach compared to the anterior approach, and, finally, by the use of patient-controlled intravenous analgesia for postoperative pain management.

There are several limitations to the present study; the retrospective design of the study limited the available data to the information contained within the clinical charts. Furthermore, the sample size was small yet comparable to that of similar research papers available in the literature. The peripheral nerve blocks were performed by different anesthesiologists, introducing a possible bias. In order to limit this problem, the blocks were performed only by consultant-level anesthesiologists and not by trainees.

## 5. Conclusions

In conclusion, PENG + LFCN Block could offer a higher MRC score compared to the FIC Block, thus allowing for prompt postoperative mobilization and physical therapy. Furthermore, less rescue oral opioids were required for the PENG group, hinting toward a possibly greater opioid-sparing effect of this block compared to the FIC Block. The results of this study may justify a larger prospective and possibly randomized trial.

## Figures and Tables

**Table 1:** Baseline characteristics of patients.

	PENG–LFCN (n = 57)	FICB (n = 23)	*p*
Age (year)	71 ± 11	68 ± 10	0.2712
Weight (kg)	74 ± 17	78 ± 11	0.2213
Height (cm)	166 ± 10	165 ± 8	0.8029
BMI (kg/m^2^)	26.6 ± 5.1	28.6 ± 3.6	0.0886
Sex, M (n/%)	27 (47.4)	12 (52.2)	0.697
ASA status	2 [2; 3]	2 [2; 3]	0.3518

Legend. PENG: Pericapsular Nerve Group. LFCN: Lateral Femoral Cutaneous Nerve. FICB: Fascia Iliaca Compartment Block. BMI: body mass index. ASA status: American Society of Anesthesiologists Physical Status Classification System.

**Table 2:** Pain control at different time points.

	PENG (n = 57)	FIB (n = 23)	*p* (Time)	*p* (Block)	*p* (Interaction)
			0.0006	0.0010	0.4301
NRS 6 h	1 [0; 2]	2 [1; 4]			
NRS 12 h	2 [1; 4]	2 [0; 5]			
NRS 24 h	1 [1; 2]	1 [0; 3]			

Legend. PENG: Pericapsular Nerve Group. NRS: Numeric Rating Scale.

**Table 3:** Difference (mean differences) in NRS when comparing FIB and PENG groups.

	FIB–PENG	95% CI
NRS 6 h	0.93	0.14; 1.72
NRS 12 h	0.47	−0.49; 1.43
NRS 24 h	0.39	0.25; 1.02
Total dose of opioids	42.5	30.8; 54.4

Legend: mean differences, expressed as FIB–PENG (positive values indicate higher mean values in the FIB group compared to the PENG group). PENG: Pericapsular Nerve Group. NRS: Numeric Rating Scale. CI: confidence interval.

## Data Availability

Data will be made available upon reasonable request to the corresponding author.
